# Comment on “Treatment of retroperitoneal sarcoma in Germany between 2000 and 2022: a retrospective analysis from the German Cancer Registry Group”

**DOI:** 10.1007/s00432-026-06591-w

**Published:** 2026-10-01

**Authors:** R. Saravanan, Paramanantham Madhavan, R. Sivayogana, K. Anusha

**Affiliations:** 1 Department of Orthopaedics, Meenakshi Medical College Hospital and Research Institute, Meenakshi Academy of Higher Education and Research, Enathur, Kanchipuram, Tamil Nadu 631552 India; 2 Department of Dermatology, Meenakshi Medical College Hospital and Research Institute, Meenakshi Academy of Higher Education and Research, Enathur, Kanchipuram, Tamil Nadu 631552 India; 3 Meenakshi College of Pharmacy, Meenakshi Academy of Higher Education and Research, Kanchipuram, Tamil Nadu India

**Keywords:** Retroperitoneal sarcoma, Cancer registry, Multivisceral resection, Registry completeness, Surgical oncology, Survival analysis, Rare cancers, Clinical epidemiology

## Abstract

The nationwide registry analysis by Beck et al. provides valuable insights into treatment patterns for retroperitoneal sarcoma in Germany over two decades, highlighting increasing use of multivisceral resection and improved survival analyses. While the study establishes an important framework for evaluating rare cancers using registry data, several methodological issues may influence the interpretation of the reported temporal trends and clinical conclusions. First, the observed increase in multivisceral resection may partly reflect progressive improvements in registry documentation and procedure coding rather than a true change in surgical practice. Second, substantial missing data for tumor grade and metastatic status raise concerns regarding potential selection bias in survival analyses performed on complete cases without sensitivity analyses. Addressing documentation completeness and the impact of missing data would strengthen confidence in the reported findings and improve the applicability of registry-based evidence for clinical decision-making and future rare cancer research.

Dear Editor,

We read with great interest the study by Beck et al. (2000), describing the treatment patterns for retroperitoneal sarcoma in the German Cancer Registry.

## Study design and interpretive framework

The reported increase in multivisceral resection, from approximately 12 percent of cases in 2001 to approximately 50 percent in recent years, is presented as evidence of a genuine national shift toward guideline-concordant surgical practice for liposarcoma. However, this conclusion sits alongside the paper's own separate observation that the rise in annual case numbers over the same period reflects improving case ascertainment and documentation completeness following the 2014 legal framework for cancer registration, rather than a true increase in disease incidence. The organ resection analysis draws on a subset of only 1318 patients with codeable resection procedures, which is considerably smaller than the full surgical cohort, and depends entirely on whether individual Operationen- und Prozedurenschlüssel codes for each resected organ were captured in the registry record. If procedure-level documentation completeness improved over the same two decades as overall case ascertainment, a plausible and untested alternative explanation is that earlier multivisceral resections were simply underdocumented, with only the single most prominent procedure code recorded, while later cases benefited from more complete coding practices that captured every organ removed during the same operation (Behrman et al. 2025). Under this explanation, part or all of the observed temporal trend would reflect improved documentation rather than a genuine shift in surgical extent, a distinction with direct consequence for the paper's central claim that registry data confirm evolving adherence to multivisceral resection guidelines over time (McGahan et al. 2026). Reporting the proportion of surgical cases with at least one codeable resection procedure by diagnosis year, alongside the current organ-count trend, would allow readers to judge whether the apparent rise in multivisceral resection tracks improving procedure documentation or reflects an independent clinical practice change (Gudmundsdottir et al. 2025). This distinction matters beyond methodological tidiness: if the trend is substantially a documentation artifact, then the paper's implicit message to clinicians and registry stewards, that German practice has progressively converged on international multivisceral resection guidelines for liposarcoma, would be overstated, and the more accurate takeaway would concern the maturation of registry coding rather than the maturation of surgical decision-making (Grant and Engelman 2025).

### Statistical validity and clinical interpretation

Grading information was missing in twenty-four percent of cases and distant metastasis status was missing in fifty-five percent, yet both variables were used to stratify the survival analyses and the primary treatment description without a sensitivity analysis addressing whether this missingness is informative. If earlier-era cases, which the paper's own discussion acknowledges suffered from less complete documentation, are disproportionately represented among those with missing grading or staging data, the survival curves and treatment-pattern tables restricted to complete cases could systematically overrepresent more recent, better-documented tumors, which may differ in surgical approach, tumor biology ascertainment, or case mix from the excluded, less-documented cases (Walsh 2025). This concern applies specifically to the Kaplan–Meier estimates stratified by grade, since these are calculated only among the roughly three-quarters of cases with recorded grading, without any comparison of baseline characteristics between graded and ungraded cases to establish that the two groups are otherwise similar (Wiegers et al. 2025). Reporting whether missingness in grading and metastasis status varies systematically by diagnosis year and providing a basic comparison of age, sex, and histology distribution between complete and incomplete cases would allow readers to judge whether the survival and treatment-pattern findings generalize to the full registry cohort or are specific to the more thoroughly documented subset (Müller et al. 2025). Given that the paper elsewhere treats improving documentation completeness as a plausible explanation for other temporal patterns in the dataset, extending that same reasoning explicitly to the grading and staging variables underlying the survival analysis would bring methodological consistency to how documentation quality is treated across the different results sections of the paper (Hong et al. 2025).

## Clinical applicability and individualized strategy

Neither concern displaces the paper's substantial contribution in establishing a reproducible, histology- and location-specific framework for extracting a rare tumor cohort from national registry data, a genuinely useful methodological template for future rare-cancer registry research beyond retroperitoneal sarcoma specifically. Distinguishing genuine practice change from improved documentation completeness in the multivisceral resection trend and characterizing whether missingness in grading and staging correlates with the diagnosis era would allow clinicians and health-policy readers to rely on the reported trends as evidence of evolving surgical practice with greater confidence rather than treating documentation artifacts as clinical signals (Shu et al. 2026).

## Data Availability

No datasets were generated or analysed during the current study.
